# The Establishment of a Novel In Vitro System for Culturing *Cytauxzoon felis*

**DOI:** 10.3390/pathogens13070565

**Published:** 2024-07-04

**Authors:** Pabasara Weerarathne, Mason Reichard, Craig Miller, Ruth C. Scimeca

**Affiliations:** Department of Veterinary Pathobiology, College of Veterinary Medicine, Oklahoma State University, Stillwater, OK 74078, USA

**Keywords:** *Cytauxzoon felis*, in vitro, cell culture, inhibition, AAE2, ISE6

## Abstract

Cytauxzoonosis, a highly fatal tick-borne disease in domestic cats caused by *Cytauxzoon felis*, poses diagnostic and therapeutic challenges due to the inability to culture the parasite in vitro. This study aimed to artificially replicate *C. felis* infection and characterize in vitro replication kinetics. Concanavalin A-activated feline embryonal macrophages (Fcwf-4) were plated at 3–5 × 10^5^ cells/mL and incubated with *C. felis*-positive blood samples from either a (1) chronically infected bobcat (*Lynx rufus*), (2) chronically infected domestic cat, or (3) acutely infected domestic cat with clinical signs of cytauxzoonosis. Temporal changes in parasite load were quantified by droplet digital PCR (ddPCR), and the inhibition of infection/replication was assessed using atovaquone, imidocarb dipropionate (ID), artemisinin, ponazuril, and neutralizing antibodies. Tick cell lines AAE2 and ISE6 were also tested for infection. In vitro inoculation with chronic infection led to transient replication, while acute infection resulted in sustained replication beyond 10 days post-inoculation. Atovaquone, ID, and artemisinin inhibited replication, and neutralizing antibodies prevented infection. The inoculation of tick cells in vitro indicated infection; however, parasite replication was not observed. The results of this study established an in vitro model for studying infection dynamics, assessing therapy efficacy, and testing vaccination strategies in cytauxzoonosis-infected cats.

## 1. Introduction

Cytauxzoonosis is a fatal tick-borne disease prevalent in domestic cats from the southeastern, south-central, and mid-Atlantic United States (US) [1,2]. When it was first discovered in Missouri in 1976, it was thought to be caused by a *Cytauxzoon*-like agent [3]. However, subsequent studies identified the causative agent as *Cytauxzoon felis*, an apicomplexan parasite belonging to the family *Theileridiae* [4,5]. The life cycle of *C. felis* is complex and completed in two hosts: sexual reproduction in an arthropod host and asexual reproduction in a vertebrate host [1,6]. Studies have identified *Amblyomma americanum* and *Dermacentor variabilis* as tick vectors [7,8,9,10], and domestic and wild felids serve as vertebrate hosts [5,11]. Infection in domestic cats typically results in severe clinical illness with rapid disease progression associated with the schizogonous phase of the parasite [1,12,13]. Thus, domestic cats are considered aberrant hosts of *C. felis* with schizont-laden macrophages acting as thrombi, resulting in vascular occlusion in vital organs and cardiovascular collapse [2,14]. Mortality rates in cats presented to veterinary clinics vary between 40 and 100% [15], and cats surviving cytauxzoonosis may serve as chronic carriers, thereby increasing the spread of the disease [13,16].

Experimental methods to study *C. felis* are limited and have impeded progress in the fight to cure cytauxzoonosis. Due to the complex life cycle of *C. felis*, attempts to culture the parasite in vitro have not been successful, hindering the development of new treatments and effective immunization strategies. As a result, there is currently no vaccine available to prevent *C. felis* infection, and attempts to develop effective antiprotozoal therapies have been met with limited success. The current standard of care (atovaquone + azithromycin) has a 60% success rate but only if infected cats are treated at an early stage of infection [17]. In vivo infection remains the only experimental model to evaluate the pathogenesis of *C. felis* infection in domestic felids, yet the practicality of in vivo studies on cytauxzoonosis are unsustainable due to high cost, ethical concerns associated with infection protocols, and the lack of funding for companion animal studies. As such, developing an in vitro cell culture system would be beneficial in studying the parasite and developing novel therapeutics for cytauxzoonosis.

The objective of the present study was to evaluate the capacity of a novel cell culture system to reproduce the life cycle of *C. felis* in vitro. Potential applications for an in vitro cell culture system are extensive and include the following: (i) the development of new and/or improved diagnostic assays for the early detection of *C. felis* infection, (ii) an evaluation of novel vaccine candidates to prevent infection, and (iii) efficacy screening for new pharmacologic agents or treatment combinations to improve therapeutic efficacy. The broad applicability of this model system will not only provide new tools in the fight against this deadly feline disease but will also contribute to the relief of animal distress by significantly reducing the need for in vivo models to study cytauxzoonosis.

## 2. Materials and Methods

### 2.1. Cell Culture

Feline embryonic macrophages (Fcwf-4; CRL-2787) (ATCC, Manassas, VA, USA) were commercially obtained and maintained in Eagle’s minimum essential medium (EMEM) (ATCC, Manassas, VA, USA) supplemented with 10% fetal bovine serum (FBS) (HyClone, Logan, UT, USA) and 1% penicillin/streptomycin (Gibco, Thermofisher Scientific, Waltham, MA, USA) at 37 °C with 5% CO_2_. Embryonal cell lines of *Amblyomma americanum* (AAE2; obtained from Departmentof Entomology, University of Minnesota) and *Ixodes scpularis* (ISE6; obtained from co-author RCS’s cell bank, Oklahoma State University) were maintained in L15B300 medium supplemented with 10% FBS and 1% penicillin/streptomycin at 34 °C without CO_2_ [18].

### 2.2. Preparation of Inoculums

Blood samples from domestic cats displaying clinical signs suggestive of acute *C. felis* infection were obtained in EDTA from licensed veterinarians in Oklahoma and Arkansas. Blood samples from cats with chronic *C. felis* infection were collected in EDTA from subclinically infected donor cats housed at the Oklahoma State University Animal Resource Facility on IACUC-approved protocols. Blood samples obtained from bobcats were collected in EDTA from specimens that were legally hunted within the state of Oklahoma. *Cytauxzoon felis* infection was confirmed in all samples prior to use in cell culture experiments via the droplet digital PCR (ddPCR) assay described in Section 2.7.

To prepare lysed blood samples for the inoculation of Fcwf-4 cells, *C. felis*-positive blood samples (500 µL) were rapidly freeze-thawed (at −80 °C and 37 °C) two times, and 200 µL of sterile H_2_O was added and allowed for the complete lysis of red blood cells. To isolate peripheral blood mononuclear cells (PBMCs), whole blood was diluted by adding phosphate-buffered saline (PBS; Fisher Scientific, Hampton, NH, USA) at a 1:1 ratio in a 15 mL tube. An equal volume of room temperature Histopaque-1077 (Sigma-Aldrich, St. Louis, MO, USA) was underlaid, and the mixture was centrifuged at 1750 rpm without a brake for 30 min. PBMCs that accumulated at the interface were collected and washed with PBS + 0.1% bovine serum albumin three times, then centrifuged at 1200 rpm for 10 min at 4 °C.

To isolate merozoites for the inoculation of AAE2 and ISE6 cells, methods described in Sugimoto et al. [19] were used with a few modifications. Briefly, 500 µL of *C. felis*-positive blood from a chronically, subclinically infected cat was mixed with 3 mL of PBS and then gently overlayered onto 5 mL of Ficoll (GE HealthCare, Chicago, IL, USA) in a 15 mL tube and centrifuged at 1750 rpm for 45 min without a brake. The supernatant was removed, and the erythrocyte pellet was mixed with 9 volumes of NH_4_Cl (0.83% NH_4_Cl in 0.17 M Tris buffer) and incubated for 3 min at 37 °C. It was then centrifuged at 15,000× *g* for 10 min at 4 °C. The supernatant was removed, and the pellet was resuspended in 1 mL of 10 mM Tris, 150 mM NaCl, and 5 mM EDTA buffer. The mixture was overlayed gently onto 2 mL of Ficoll in a 15 mL tube and centrifuged at 3600× *g* for 20 min at 4 °C. The second layer from the top was collected, washed two times with Tris-NaCl-EDTA (15,000× *g* for 10 min at 4 °C), and the pellet was resuspended in 200 µL of complete cell culture media.

### 2.3. Experimental Infection of Fcwf-4 Cells with C. felis-Positive Blood

**Figure 1:**
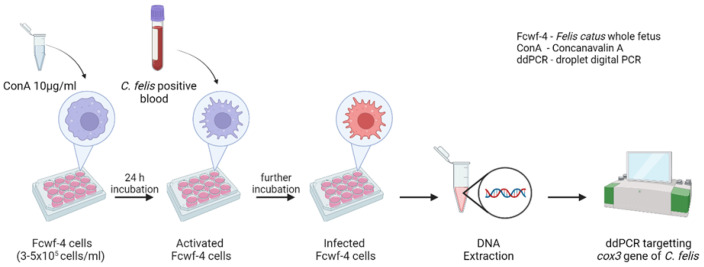
A schematic diagram of the procedure followed to inoculate Fcwf-4 cells with *C. felis*-positive blood and collect samples to quantify parasite load using ddPCR. Created with BioRender.com.

Fcwf-4 cells were plated in 24- or 48-well plates at 3.0–5.0 × 10^5^ cells/mL in duplicate with 10 µg/mL Concanavalin A (ConA) (Sigma-Aldrich, St. Louis, MO, USA). Cells were incubated for 24 h at 37 °C and 5% CO_2_ to facilitate the activation of feline macrophages. Following incubation, *C. felis*-positive lysed blood or whole blood from a (i) bobcat, (ii) chronically infected domestic cat, or (iii) acutely infected domestic cat was added (12.5 µL) to the cells. Cells without the addition of blood served as controls. Cells were incubated for up to 10 days at 37 °C and 5% CO_2_. Cell samples were collected on 0, 2, 4, 6, 8, and 10 days post-infection (dpi) to quantify the parasite load in each cell sample via ddPCR (Figure 1 and Section 2.7). The collected cell samples were centrifuged at 13,000 rpm for 15 min, and the cell pellets were stored at −20 °C until DNA extraction (Section 2.6).

### 2.4. Experimental Infection of AAE2 Cells with C. felis Merozoites

**Figure 2:**
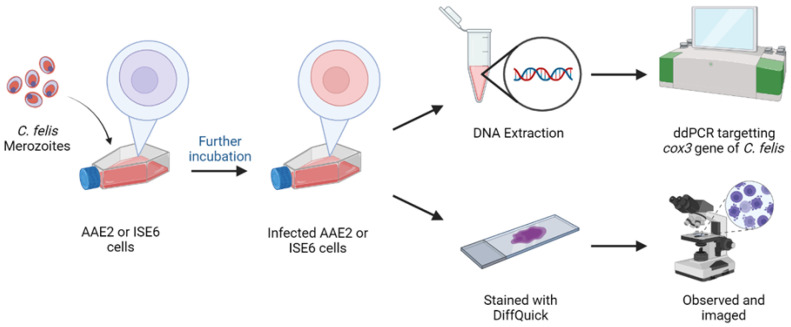
A schematic diagram of the procedure followed to inoculate AAE2 and ISE6 cells with *C. felis* merozoites and collect samples to quantify parasite load using ddPCR and to capture cell images. Created with BioRender.com.

AAE2 cells were plated at 5.0 × 10^5^ cells/mL in 12-well plates in duplicate and allowed to reach 90–100% confluency. Isolated merozoites (as described in Section 2.2) were added (12.5 µL) to the wells and incubated for up to 12 days at 34 °C without CO_2_. Cell samples were collected on 0, 2, 4, 6, 8, 10, and 12 dpi to quantify the parasite load in each cell sample (Section 2.7). Additionally, 90–100% confluent AAE2 and ISE6 cells in T-25 culture flasks (Corning Life Sciences, Durham, NC, USA) were inoculated with 50 µL of merozoite suspension and incubated at 34 °C. Cell samples were collected on 4 and 7 dpi. A portion of these cell samples was centrifuged at 1000 rpm for 5 min, followed by the removal of the supernatant. Cells were fixed on glass slides and stained via DiffQuick staining (EKI-chem, Joliet, IL, USA). Stained cells were observed under light microscopy for evidence of *C. felis* infection. The remaining portion of cells was used to extract DNA for ddPCR (Figure 2).

### 2.5. In Vitro Inhibition of C. felis

Fcwf-4 cells were plated as described in Section 2.2. However, before the addition of lysed *C. felis*-positive blood, ponazuril (Sigma-Aldrich, St. Louis, MO, USA), artemisinin (Sigma-Aldrich, St. Louis, MO, USA), or imidocarb dipropionate (Sigma-Aldrich, St. Louis, MO, USA) was added to duplicate cultures to achieve final concentrations as indicated in Table 1, at 48 h intervals for a duration of up to 10 days. Atovaquone (Sigma-Aldrich, St. Louis, MO, USA) was added only on day 0 without a supplementation. To test neutralizing antibody activity, plasma from a cat with acute cytauxzoonosis having high levels of anti-*C. felis* antibodies was used. Anti-*C. felis* antibodies were quantified as previously described [20]. Plasma (50 µL) was added to duplicate cultures of cells prior to inoculation with lysed blood. All treated/infected cell cultures were incubated and sampled as described in Section 2.2. The culture media added to the cells at the beginning of the experiment remained unchanged throughout the study.

### 2.6. DNA Extraction

Following the collection of cells at each sampling point, cell mixtures were spun at 13,000 rpm for 15 min. The supernatant was removed, and DNA was extracted from the cell pellets using the DNeasy^®^ Blood & Tissue DNA extraction kit (Qiagen, Valencia, CA, USA) per the manufacturer’s instructions. DNA was eluted into 50 μL of elution buffer, and the quality and quantity of the extracted DNA were measured using a NanoDrop 2000 Spectrophotometer (Thermo Fisher Scientific, Waltham, MA, USA). Samples were stored at −20 °C until used for ddPCR.

### 2.7. Parasite Quantification

Parasite load was quantified using a probe-based ddPCR method as previously described [21] with a few modifications. Briefly, a mixture consisting of 13.3 μL of ddPCR master mix (created by blending 12 μL of super mix for probes without dUTP, 1.2 μL of primer/probe mix, and 1.2 μL of DNase-free water) was combined with 8.8 μL of DNA samples (containing equal amount of DNA) prepared in Section 2.6 to generate the reaction mixture. An aqueous droplet-in-oil mixture was prepared by mixing 20 μL of the reaction mixture with droplet generation oil for probes (no dUTP) (Bio-Rad Laboratories, Inc., Hercules, CA, USA). A total of 40 μL of this final mixture was added to a 96-well plate, sealed, and then PCR was conducted using a C1000 Touch™ Thermal Cycler (Bio-Rad Laboratories, Inc., Hercules, CA, USA) according to parameters documented in Kao et al. [21]. Following PCR amplification, the modules were moved to the QX200™ Droplet Reader (Bio-Rad Laboratories, Inc., Hercules, CA, USA) to quantitate the target DNA (*cox3* gene). All samples were tested in duplicate, including a positive control from an experimentally infected domestic cat, a negative control from a specific pathogen-free domestic cat, and a no-template control. Data analysis was performed using QuantaSoft software version 1.7. Thresholds were set within the range of values from the negative control (<1500) to the positive control (ranging from 2000 to 3000). Samples were considered positive if at least one droplet in each duplicate was classified as positive. The software calculated the absolute copy numbers per reaction based on the ratio of positive to negative droplets using the Poisson law of small numbers, as described by Hindson et al. [22].

### 2.8. Statistical Analysis

Differences in parasite load and cytokine concentration were assessed and compared over time and treatment by repeated measures ANOVA or 2-way ANOVA. *p*-values < 0.05 were considered significant. All analyses were conducted using GraphPad Prism 9.0 and 10.0 software (La Jolla, CA, USA).

## 3. Results

### 3.1. Inoculation of Fcwf-4 Cells with Chronically Infected Blood Results in Transient Infection, while Acutely Infected Blood Results in Sustained Infection

**Figure 3:**
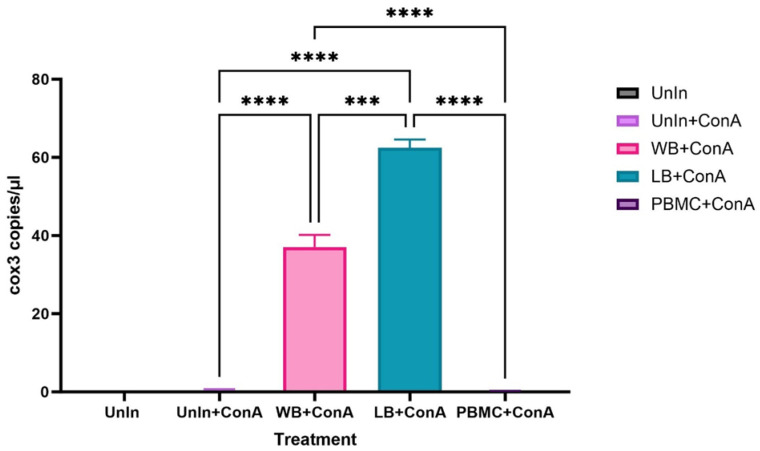
Parasite loads in Fcwf-4 cells infected with *C. felis*-positive (chronic infection) lysed blood (LB), whole blood (WB), PBMCs compared to the uninfected cells (UnIn) at 11 dpi. Parasite load in the cells infected with LB showed the highest parasite *cox3* copy numbers. *** *p* < 0.001, **** *p* < 0.0001.

To test the initial suitability of *C. felis*-positive lysed blood, whole blood, or PBMCs as an inoculum to infect feline macrophages, blood from a chronically infected cat was used to infect feline macrophages. Samples were collected at 11 dpi, and parasite load was quantified using ddPCR (Figure 3). Macrophages inoculated with *C. felis*-positive lysed blood and whole blood exhibited a significantly (*p* < 0.0001) higher parasite load compared to the control. There was no significant difference (*p* < 0.05) in the parasite load between the macrophages inoculated with *C. felis*-positive PBMCs and the control. As lysed blood showed promising results for the inoculation of macrophages, the rest of the experiments were conducted using *C. felis*-positive lysed blood.

**Figure 4:**
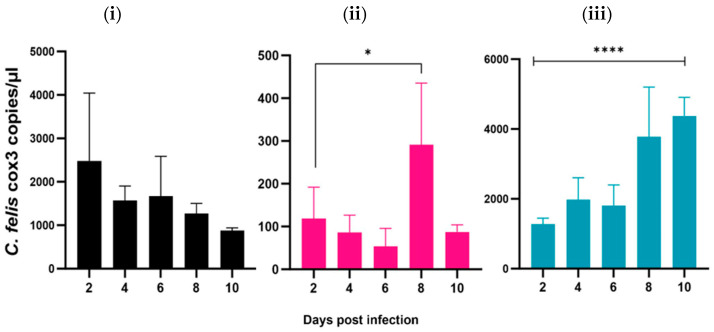
ddPCR results of in vitro experiment with blood obtained from *C. felis*-infected domestic cats or bobcat samples (acute vs. chronic infection). Feline macrophages were activated with ConA and then infected with lysed blood from either a (**i**) chronically infected bobcat, (**ii**) chronically infected domestic cat, or (**iii**) acutely infected domestic cat with *C. felis*. Cells infected with chronically infected blood with *C. felis* caused transient infection and replication within Fcwf-4 feline embryonal macrophages, but replication was not sustained. Blood from an acutely infected domestic cat with clinical signs of cytauxzoonosis caused sustained infection in activated feline embryonal macrophages that resulted in prolific parasite replication with a significant increase in *C. felis* DNA over time. * *p* < 0.05, **** *p* < 0.0001.

To determine which life stage of the parasite is the most suitable for inducing a sustained infection and parasite proliferation in vitro, three different sample types were tested: (i) lysed blood from a chronically infected bobcat, (ii) lysed blood from a chronically infected domestic cat, and (iii) lysed blood from an acutely infected domestic cat. The in vitro inoculation of stored blood samples obtained from an acutely infected domestic cat with clinical signs of cytauxzoonosis showed a significant increase (*p* < 0.0001) in the *C. felis* DNA copies over time, indicating sustained parasite replication (Figure 4). Moreover, upon staining inoculated Fcwf-4 cells with DiffQuick, distinct cell structures were observed that displayed morphological features of 1–3 µm basophilic merozoites located within schizonts (Figure S1). Compared to 2 dpi, there was a fivefold increase in the *C. felis* DNA copies by 10 dpi in those cells treated with lysed blood from a domestic cat with acute infection (Figure 4). A simple linear regression was performed to assess the time vs. parasite load interaction, and there was a significantly (*p* < 0.0001) positive correlation (Y = 399.4*X + 245.4). In contrast, *C. felis* DNA copies in macrophages that were inoculated with blood from a chronically infected bobcat decreased over time (Figure 4). Though the decrease was significant by 10 dpi compared to that in 2 dpi, there was a significant (*p* < 0.05) negative correlation between time and *C. felis* DNA copies (Y = −175.3*X + 2626). There was a slight decrease by 6 dpi; however, it was not significant (*p* > 0.05). Macrophages that were inoculated with blood from a chronically infected domestic cat resulted in a significant but transient increase in *C. felis* DNA copy numbers at 8 dpi compared to baseline (*p* < 0.05), but this was not sustained (Figure 4). There was an association between time and parasite load (Y = 7.103*X + 84.82); however, it was not statistically significant (*p* < 0.05). These results indicated that lysed blood from domestic cats with acute infection was the most suitable for further use in an in vitro model to test the efficacy and replication inhibition of antiprotozoal therapies and/or to test the effect of neutralizing antibodies. As such, Fcwf-4 cells inoculated with lysed blood from an acutely infected domestic cat were used for subsequent experiments.

### 3.2. Antiprotozoal Drugs and Neutralizing Antibodies Inhibit C. felis Replication In Vitro

**Figure 5:**
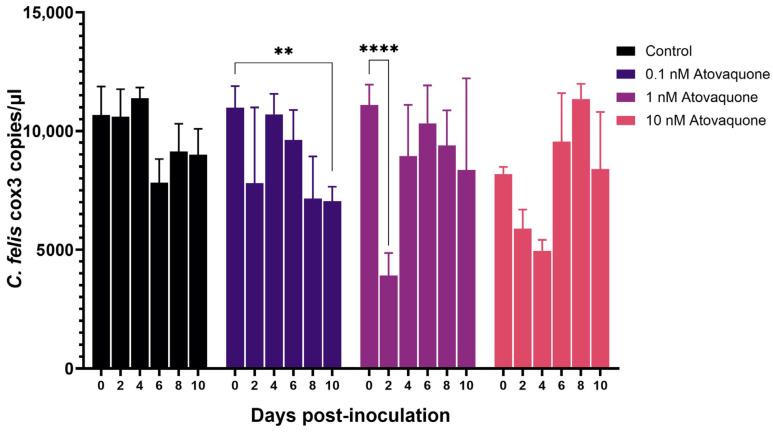
Parasite load in activated feline macrophages inoculated with lysed blood from an acutely infected domestic cat were treated with varying concentrations of atovaquone (0.1 nM, 1 nM, and 10 nM). Overall, there was a decrease in the *C. felis* DNA copies in treated groups compared to the control. Cells treated with 0.1 nM atovaquone showed a significant (*p* < 0.01) decrease in parasite load by 10 dpi. A significant decrease in *C. felis* DNA copies was observed in 10 nM-treated cells up to 4 dpi compared to the cells that were not treated, indicating the transient suppression of protozoal replication. ** *p* < 0.01, **** *p* < 0.0001.

To test the applicability of the cell culture system to test novel therapeutics and vaccine efficacy, various antiprotozoal drugs (listed in Table 1) and anti-*C. felis* antibodies acquired from a cat with acute cytauxzoonosis were used to inhibit *C. felis* infection and replication. When macrophages infected with lysed blood from an acutely infected cat were treated with 0.1 nM, 1 nM, and 10 nM atovaquone, cells treated with 10 nM atovaquone showed an overall reduction in the parasite load. *Cytauxzoon felis* DNA was significantly (*p* < 0.0001) reduced in macrophages treated with atovaquone until 4 dpi compared to the untreated control (Figure 5). However, by 6 dpi, parasite load increased in the 10 nM-treated cells compared to the control before starting to decrease by 10 dpi. In the group treated with 1 nM atovaquone, *C. felis* DNA copies were drastically (*p* < 0.0001) reduced at 2 dpi but increased again as was previously observed with the 10 nM treatment. A steady but significant reduction was observed in the cells that were treated with 0.1 nM. However, these reductions did not reach statistical significance when compared to the untreated control group.

**Figure 6:**
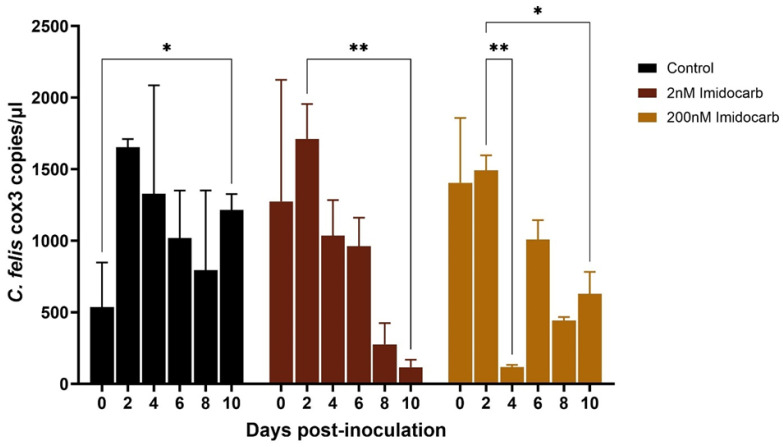
Parasite load in the activated feline macrophages infected with lysed blood from an acutely infected domestic cat with *C. felis* that were then treated with varying concentrations of imidocarb dipropionate (2 nM and 200 nM). *C. felis* replication reduced gradually over time under 2 nM treatment. With 200 nM treatment, a transient suppression of protozoal replication was observed. * *p* < 0.05, ** *p* < 0.01.

**Figure 7:**
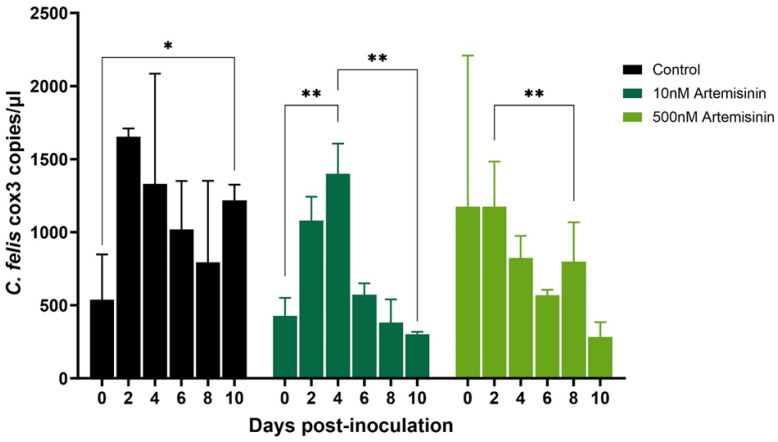
Parasite load in the activated feline macrophages infected with lysed blood from an acutely infected domestic cat with *C. felis* that were then treated with varying concentrations of artemisinin (10 nM and 500 nM). *C. felis* replication reduced gradually over time with both treatments. * *p* < 0.05, ** *p* < 0.01.

**Figure 8:**
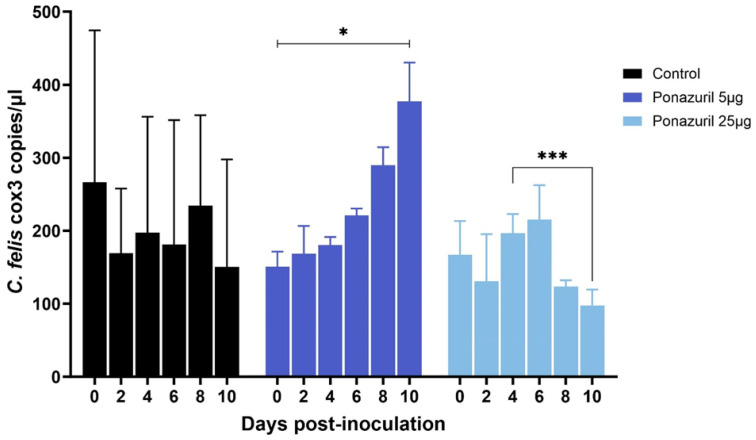
Parasite load in the activated feline macrophages infected with lysed blood from an acutely infected domestic cat with *C. felis* that were then treated with varying concentrations of ponazuril 5 µg and 25 µg. *C. felis* replication was not inhibited with 5 µg treatment; however, 25 µg showed an inhibition of parasite replication. * *p* < 0.05, *** *p* < 0.001.

**Figure 9:**
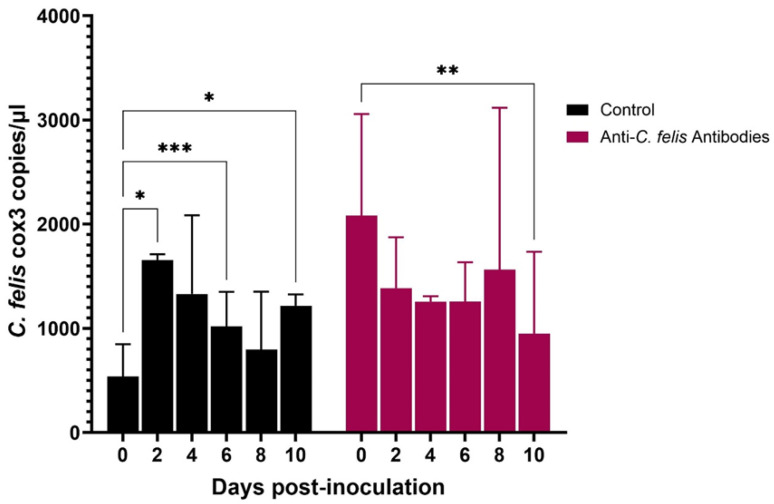
Parasite load in the activated feline macrophages infected with lysed blood from an acutely infected domestic cat with *C. felis* that were then treated with plasma from a cat with acute *C. felis* infection containing anti-*C. felis* antibodies. *Cytauxzoon felis* replication remained static and was significantly reduced by 10 dpi. * *p* < 0.05, ** *p* < 0.01, *** *p* < 0.001.

Imidocarb dipropionate was used at 2 nM and 200 nM concentrations. Compared to the untreated control, both 2 nM and 200 nM treatments showed a significant (*p* < 0.05) decrease in the parasite DNA copies over time (Figure 6). However, while macrophages treated with 2 nM imidocarb showed a gradual decrease in parasite load up to 10 dpi, the cells that received 200 nM showed a drastic decrease in parasite load by 4 dpi and increased significantly (*p* < 0.01) by 6 dpi before starting to decrease again. Treatment with artemisinin (10 nM and 500 nM) also showed a significant decrease (*p* < 0.05) in parasite load by 10 dpi (Figure 7). Similar to imidocarb, an increase in parasite load was observed until 4 dpi (for 10 nM treatment) or 2 dpi (for 500 nM treatment) before seeing a reduction in the *C. felis* DNA copies. Interestingly, treatment with 5 µg of ponazuril did not show a decrease in *C. felis* DNA copies. In contrast, a significant (*p* < 0.05) increase in parasite load was observed over time (Figure 8). However, with the 25 µg ponazuril treatment, there was a significant reduction in parasite DNA copy numbers after 6 dpi, indicating parasite inhibition. When plasma from a cat with acute *C. felis* infection was used to test for the activity of neutralizing antibodies, a significant reduction (*p* < 0.01) in parasite DNA copy numbers was observed compared to an increase (*p* < 0.05) in the untreated control group by 10 dpi (Figure 9).

### 3.3. Cytauxzoon felis Merozoites Infects AAE2 and ISE6 Cells In Vitro but Does Not Replicate

**Figure 10:**
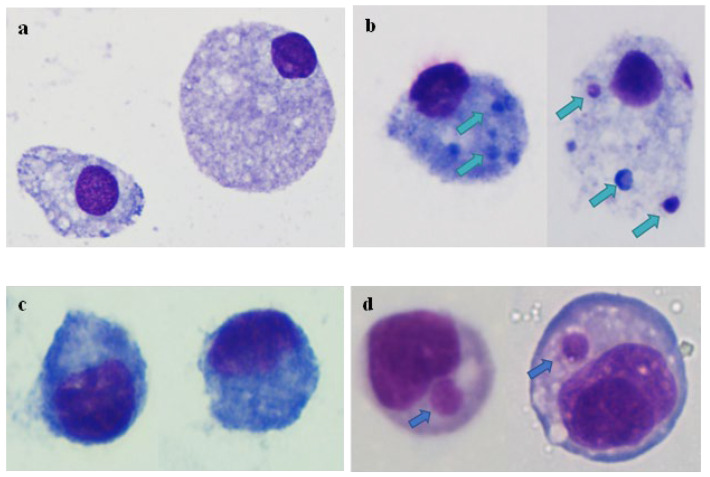
Light microscopic images of DiffQuick-stained AAE2 (**upper panel**) and ISE6 (**lower panel**) cells. Cells were inoculated with merozoites isolated from a chronically infected cat. (**a**) Uninfected AAE2 cells, (**b**) infected AAE2 cells, (**c**) uninfected ISE6 cells, (**d**) infected ISE6 cells. Arrows indicate the inclusions observed in the cells that were incubated with *C. felis* merozoites, possibly representing kinete or sporozoite life stages of *C. felis*.

**Figure 11:**
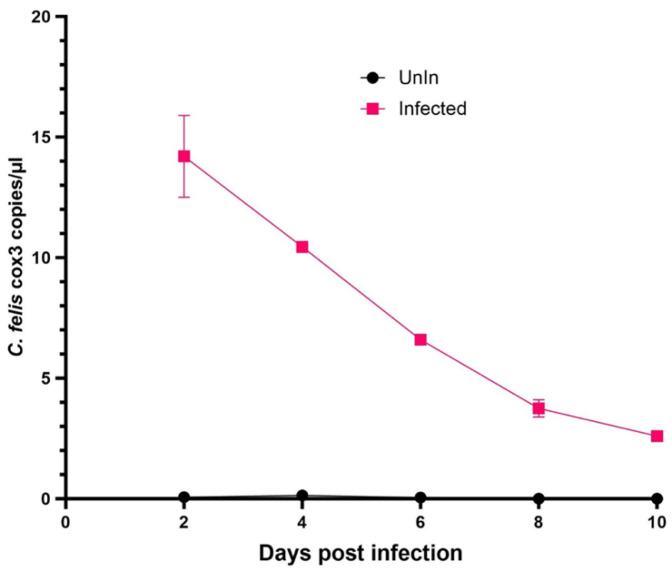
ddPCR results of the AAE2 cells infected with *C. felis* merozoites isolated from a chronically infected domestic cat. *Cytauxzoon felis cox3* copy numbers in the infected cells decreased over time. This could be attributed to several factors including the inability of the parasite to survive in the cell culture system (pH of the media, temperature, growth factors) and inability of the tick cells to properly function due to unfavorable culture conditions (i.e., growth rate in well plates).

Embryonic cells of *A. americanum* (AAE2) and *I. scapularis* (ISE6) were inoculated with merozoites isolated from blood samples of chronically *C. felis*-infected cats. Cell samples were further incubated and stained with DiffQuick to observe for any morphological indications of infection using a light microscope. Inclusions were observed in both AAE2 and ISE6 cells that were inoculated with *C. felis* merozoites compared to the cells that were not inoculated (Figure 10). *Cytauxzoon felis*-specific *cox3* copies were initially detected at 2 dpi in these cells by ddPCR; however, *cox3* copy numbers steadily decreased over time with no evidence of replication (Figure 11).

## 4. Discussion

In vitro models are used extensively for studying pathogenic mechanisms and for the development of therapeutics and diagnostics. Several in vitro models are available for apicomplexan parasites that are important causes of diseases in animals and humans. Particularly for tick-borne diseases, in vitro models play a crucial role, as in vivo studies can be labor-intensive, expensive, and challenging from the point of view of ethics. Unfortunately, for cytauxzoonosis, attempts to develop an in vitro model and replicate the parasite in cell culture have not been successful to date [23,24], and in vivo models remain the only available option [9].

In the present study, we activated feline embryonal macrophages with Concanavalin A (ConA) and infected them with *C. felis*. Preliminary studies by our lab showed that Fcwf-4 cells activated with ConA were 100 times more likely to become infected with *C. felis* compared to the cells that were not activated (Figure S2). Consistent with these findings, several studies have shown that ConA enhances the phagocytosis of macrophages [25,26,27], which might be playing an important role in *C. felis* infection. We further assessed the optimal sample type for infectivity, revealing that the infection of Fcwf-4 cells with lysed blood from an acutely infected cat with *C. felis* yielded compelling results. Notably, our observations included intracellular accumulations of 1–3 µm, basophilic structures morphologically resembling merozoites, and a statistically significant (*p* < 0.0001) increase in *C. felis* DNA copies over time, demonstrating a remarkable fivefold rise by the 10th day post-infection compared to the baseline at day 0 (Figure 4). This robust increase in *C. felis* DNA copies that sustained for a period of 10 days strongly supports the notion that the parasite replication is taking place within our cell culture system. While the significant increase (*p* < 0.05) in parasite copies observed in the case of blood samples from a chronically infected domestic cat at 8 dpi suggests the possibility of transient infection and replication within Fcwf-4 feline embryonal macrophages, it is crucial to note that this replication was not maintained beyond 8 dpi. Similarly, though not significant, a slight increase in the parasite DNA copies was observed at 6 dpi in the cells inoculated with blood from a bobcat. However, the steady decrease in *C. felis* DNA copy numbers in cells after 6 dpi indicates that the parasite either fails to infect or replicate and thrive within the cell culture system. These findings underscore the unique dynamics observed with different blood samples, with the acutely infected blood showing the most robust and sustained replication, followed by the samples from chronic infection.

The variability in outcomes observed in this experiment may be attributed to the different life stages of *C. felis* present in the blood samples. In the chronic phase, merozoites are the predominant life stage, residing within erythrocytes, where they continuously divide asexually and reinfect red blood cells rather than macrophages [1,2,4]. While the results of this study indicate that the life stage associated with chronic infection (merozoite) may be suitable to evaluate infection kinetics in vitro, these infection sources may not be ideal for longer-term in vitro studies or for propagating *C. felis*. In contrast, acutely infected blood contains schizont-laden macrophages and possibly sporozoites, the infectious life stage of *C. felis* to felids [2]. It is important to note that, during the initial stages of infection, the parasites within mature schizont-laden macrophages may be actively released through schizont rupture, subsequently reinfecting neighboring macrophages. This phenomenon is further supported by the observed increasing pattern in *C. felis* DNA copy numbers within cells inoculated with blood from the acute phase of infection. The marked difference in infection dynamics between these blood groups underscores the significance of understanding the varying life stages of the parasite and their implications for in vitro studies and propagation.

Our studies have yielded promising results regarding the replication of *C. felis* within the cell culture system. To validate the potential use of this in vitro model, we conducted experiments using established antiprotozoal drugs and *C. felis*-neutralizing antibodies to inhibit parasite replication and infection. The results clearly demonstrated significant (*p* < 0.05) reductions in *C. felis* DNA copies when treated with various antiprotozoal agents and neutralizing antibodies, providing evidence that the cultivation of *C. felis* using this system is indeed feasible and holds potential for developing therapeutics or prophylaxes.

Atovaquone is the antiprotozoal drug that is currently being used to treat patients with acute cytauxzoonosis. Early detection and treatment with atovaquone together with azithromycin and supportive care have shown a 60% survival rate in infected cats [17]. Atovaquone is a structural analog of protozoan ubiquitin and acts by inhibiting the binding of ubiquitin to cytochrome b and thereby inhibiting the electron transport chain [28,29]. In the present study, and consistent with in vivo findings, we saw a reduction in *C. felis* DNA copies indicating parasite inhibition. However, the inhibition that was seen in the cells treated with 1 nM and 10 nM was transient, and the parasite load started to increase beyond 4 dpi. However, these cells did not receive a supplementation of atovaquone during the 10-day period of this study. As such, the increase in the parasite load after 4 dpi is likely due to degradation and the decrease in the concentration of atovaquone present in the cell culture media. An additional supplementation of atovaquone and/or higher concentrations of the drug may likely result in sustained inhibition.

The other antiprotozoal drugs tested in the present study were imidocarb dipropionate, artemisinin, and ponazuril. Imidocarb dipropionate is an antiprotozoal used for treating large-sized *Babesia* species infections [30]. It inhibits protozoans by interfering with polyamine production [31] and/or by damaging the nucleic acid of the parasite, thereby inhibiting cellular repair and replication [32]. Imidocarb has been used to treat cytauxzoonosis prior to the identification of atovaquone being more effective. With imidocarb, only a 26% survival rate has been reported in cats infected with cytauxzoonosis [17]. Additionally, imidocarb was known to be associated with adverse reactions, worsening the prognosis [33]. When we tested imidocarb in vitro, the parasite load significantly decreased over time indicating parasite inhibition (Figure 6). This was more evident in the cells that received 2 nM imidocarb at 48 h intervals. However, when cells were treated with 200 nM imidocarb, there was a drastic reduction in the parasite load by 4 dpi; this reduction was transient, with a significant increase at 6 dpi. It is possible that high doses of imidocarb interfere with host cellular function giving an advantage for the parasite to subsequently replicate.

Similar to imidocarb, artemisinin also inhibited parasite replication by 10 dpi (Figure 7). Even though an increase in the *C. felis cox3* DNA copies were observed up to 4 dpi, the continuous supplementation of artemisinin (every 48 h) resulted in a significant decrease in the parasite copy numbers, indicating the inhibition of parasite replication. Artemisinin is a widely used drug to treat malaria infections worldwide [34], and upon activation, it induces the destruction of parasite proteins through free radical production [35]. Artemisinin has a very short half-life [36]; therefore, the increase in *C. felis cox3* copy numbers at 4 dpi could be attributed to that. However, continuous supplementation may have facilitated the subsequent reduction in *C. felis cox3* DNA copies. The results of the present study suggest the potential of using artemisinin to treat cats with acute cytauxzoonosis.

Ponazuril is an anticoccidial drug used to treat equine protozoal myeloencephalitis caused by *Sarcocystis neurona* [37,38]. It most likely acts on the apicoplast of the parasite [39]. A study by Mitchell et al. [40] suggested that ponazuril can also act differently on various apicomplexan parasites by targeting different enzyme/enzyme systems and pathways. A recent study has successfully used ponazuril (30 mg/kg orally every 24 h) with azithromycin to treat against experimental infection with *C. felis* [41]. However, in the present study, in vitro treatment with ponazuril did not inhibit parasite replication (Figure 9). There was a reduction in the parasite load observed at 8 dpi and 10 dpi with 25 µg/mL treatment; however, the high concentration of ponazuril was toxic to the host cells, causing cell death, which could have contributed to the decrease in parasite copy numbers. Reagent-grade ponazuril was dissolved in DMSO [37], and in the present study, dissolved ponazuril was added to the cells in a way that the final DMSO percentage would be 0.01% as described previously [37]. However, Fcwf-4 cells are very sensitive, and the presence of DMSO might have led to adverse effects on the host cells, making it more advantageous for parasite replication rather than inhibition. In addition, during in vivo infection, the mechanism by which the drug inhibits parasite growth can be affected by several factors including host immune response, which is lacking in an in vitro system. This might explain the discrepancies observed in the present study when compared to the study by Yang et al. [41].

Previous studies on cytauxzoonosis have shown that infected cats develop a very high antibody response for *C. felis* infection [41,42,43]. Interestingly, when plasma from a domestic cat acutely infected with *C. felis* was added to Fcwf-4 cells prior to inoculating with *C. felis*, the parasite copy numbers remained static until 8 dpi and significantly reduced by 10 dpi compared to 0 dpi (Figure 9). This suggests that anti-*C. felis* antibodies present in the plasma prevented the parasite from replicating at full potential in Fcwf-4 cells. Collectively, the findings of the present study show the possibility of using the in vitro system for screening neutralizing antibodies for developing therapeutics and diagnostics and for testing the efficacy of antiprotozoal drugs against *C. felis* infection.

Tick cell lines have been used to study tick-borne diseases [44], to prepare antigens for vaccination [45], for pathogen propagation [46,47,48,49,50], to understand host-pathogen-vector interactions in a controlled environment [51,52], and for disease control [53]. Therefore, by using tick cell lines AAE2, ISE6, and chronically infected blood, we attempted to replicate gametocyte/merozoite infection in vitro. The images of the inoculated tick cells showed inclusions suggesting possible infection (Figure 10), and the ddPCR results were positive for the *C. felis* cox3 gene, further supporting the observations of cell entry. However, in both AAE2 and ISE6 cells, parasite copies continued to decrease, indicating that the parasite did not replicate in vitro (Figure 11). Infected tick cells were added to activated Fcwf-4 cells in an attempt to link the tick phase with the feline phase in vitro. In feline macrophages that received infected AAE2 cells, there was a drastic decrease in the parasite load by 4 dpi. However, an increase in parasite replication was observed on day 6, indicating a transient infection that was not sustained (Figure S3). Compared to AAE2 cells, ISE6 is a widely used and well-established cell line [44]. Though our attempts to replicate the feline-tick cycle did not succeed, our findings show the potential of using ISE6 cells to replicate *C. felis* tick phase infection. As such, optimizing the ISE6 culture conditions to facilitate *C. felis* infection leading to sustained replication could potentially help link the tick phase with the feline phase.

As the present study highlights the ability to reproduce *C. felis* replication in vitro, future studies can employ different tools such as omics or single-cell RNA sequencing to systematically delineate the pathogenic mechanisms and immune activation pathways contributing to disease progression. While this system provides valuable insights into the infection dynamics and suitability for in vitro evaluation, several limitations should be acknowledged. One of the drawbacks in the present study is that the blood samples used were from different cats from different regions (Arkansas, Oklahoma). This brings variabilities in terms of the stage of the infection, parasite load, severity, and strain of the *C. felis* used. However, despite these variations, our results were consistent in showing a prolific increase in the parasite load. Additionally, the cell culture system mainly focuses on the interaction between the parasite and host cells. It does not fully encompass the complex immune responses and interactions occurring within a living host. Incorporating additional immune components or using co-culture systems that simulate the immune response could provide a more comprehensive understanding of host-parasite interactions. Lastly, sustaining *C. felis* infection in the cell culture system remains challenging beyond a certain point. Further research is needed to explore different culture conditions, media compositions, or adaptation methods to extend the period of parasite propagation, allowing for long-term infection studies.

## Figures and Tables

**Table 1:** The concentrations of the antiprotozoal drugs used for the inhibition of *C. felis* in vitro.

Antiprotozoal	Final Concentration in Cell Culture
Atovaquone	0.1 nM, 1 nM, 10 nM
Imidocarb dipropionate	2 nM, 200 nM
Artemisinin	10 nM, 500 nM
Ponazuril	5 µg/mL, 25 µg/mL

## Data Availability

The data presented in this study are contained within the article.

## Supplementary Materials

The following supporting information can be downloaded at https://www.mdpi.com/article/10.3390/pathogens13070565/s1, Figure S1: Light microscopic images of Fcwf-4 cells stained with DiffQuick. (a) Control, (b) inoculated with *C. felis*-positive blood; Figure S2: *Cytauxzoon felis cox 3* copies in the Fcwf-4 cells inoculated with *C. felis* infected blood in the presence and absence of Concanavalin A. Figure S3: The ddPCR results of the in vitro experiment on the infection of Fcwf-4 cells with AAE2-infected cells.
