# Supplementary material for: The Establishment of a Novel In Vitro System for Culturing Cytauxzoon felis
Source: Pathogens. 2024 Jul 4;13(7):565. doi: 10.3390/pathogens13070565 (PMC11279574; doi:10.3390/pathogens13070565)
Supplement: Supplementary file 1 [file pathogens-13-00565-s001.zip › pathogens-3056411-supplementary.pdf]

pathogens

MDPI

Supplementary Material

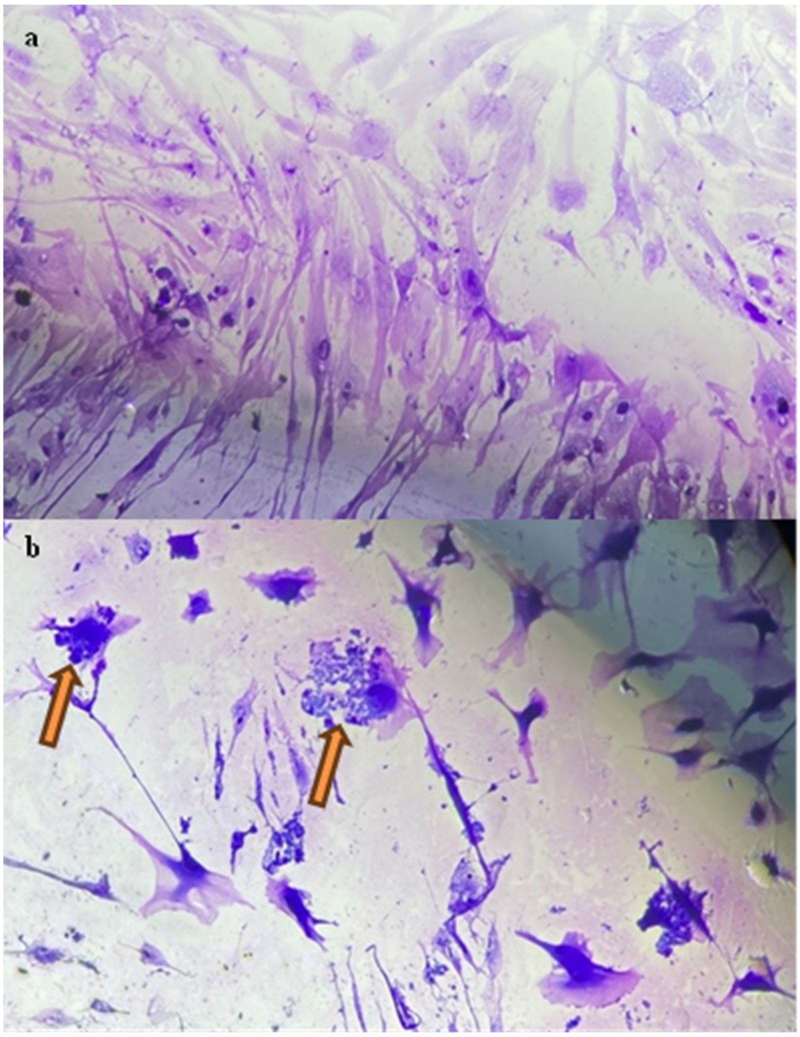

Figure S1. Light microscopic images of Fcwf–4 cells stained with DiffQuick. (a) control (b) inoculated with *C. felis* positive blood.

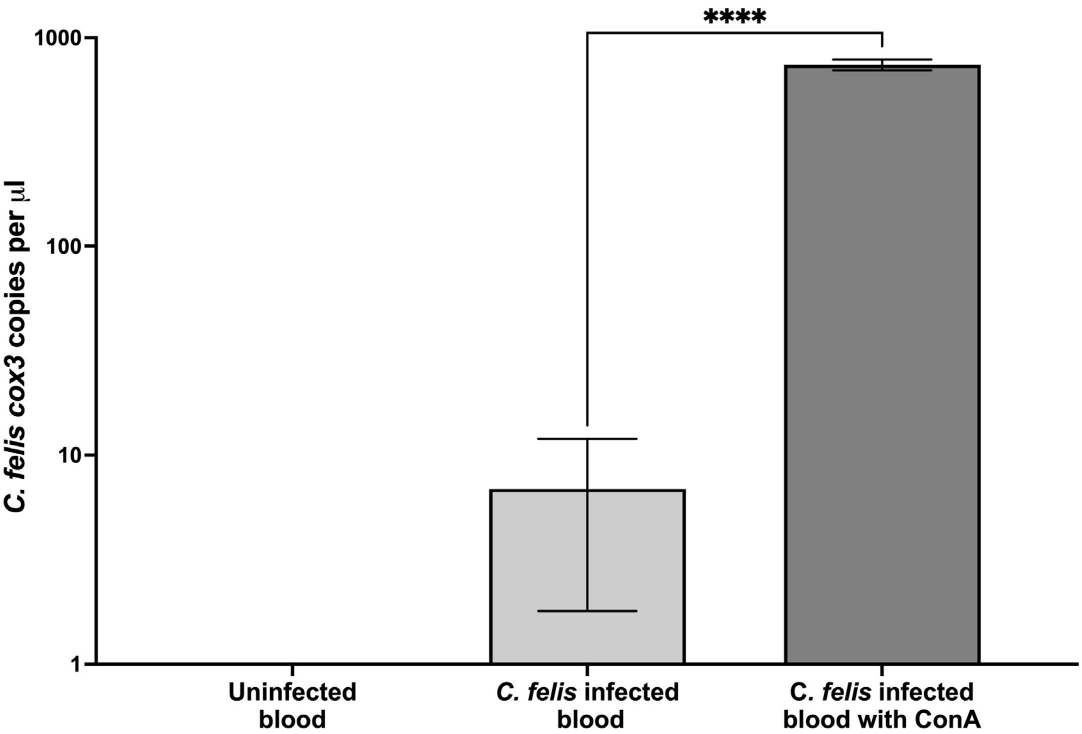

Figure S2. *Cytauxzoon felis cox 3* copies in the Fcwf-4 cells inoculated with *C. felis* infected blood in the presence and absence of Concanavalin A (ConA). Fcwf–4 cells activated with ConA were 100 times more likely to become infected with *C. felis* compared to the cells that were not activated.

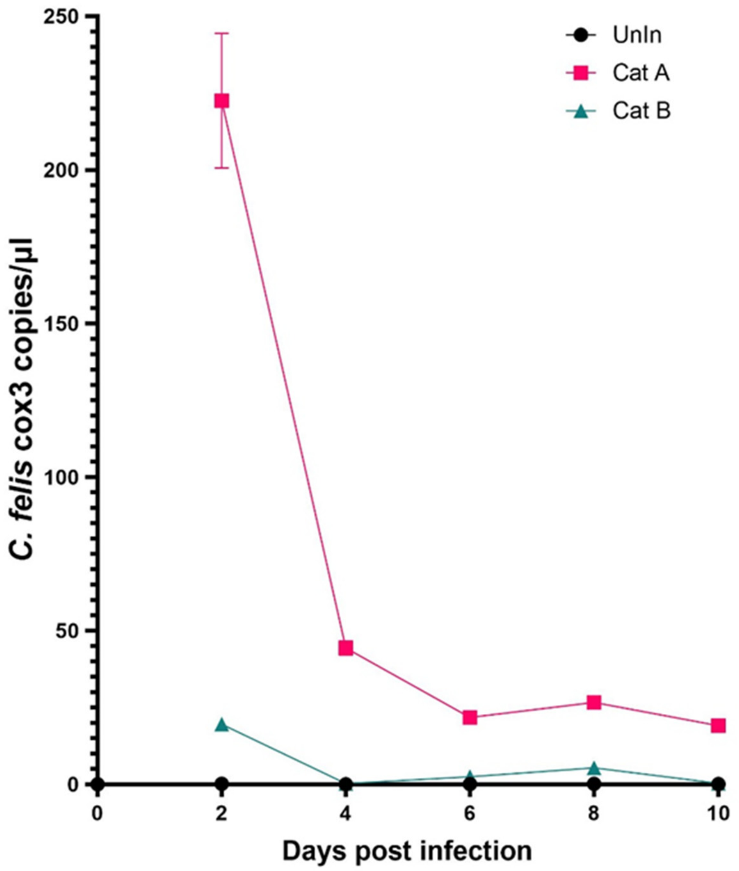

Figure S3. ddPCR results of in vitro experiment on the infection of Fcwf-4 cells with AAE2-infected cells.
